# Determinants of Z-Score of Bone Mineral Density among Premenopausal Saudi Females in Different Age Groups: A Cross Sectional Study

**DOI:** 10.3390/nu15194280

**Published:** 2023-10-08

**Authors:** Intessar Sultan, Inass Taha, Shereen El Tarhouny, Rehab A. Mohammed, Azza M. Abdu Allah, Omar Al Nozha, Maha Desouky, Abdelrahman Ghonimy, Yara Elmehallawy, Nawaf Aldeeb, Yara Ayman Iskandarani

**Affiliations:** 1 Ibn Sina National College for Medical Studies, Jeddah 22421, Saudi Arabia; shereeneltarhouny@ibnsina.edu.sa; 2 College of Medicine, Taibah University, Medina 41477, Saudi Arabia; itaha@taibahu.edu.sa (I.T.); ozoghaibi@taibahu.edu.sa (O.A.N.); mahadesouky7@gmail.com (M.D.); 3 College of Medicine, Zagazig University, Zagazig 31527, Egypt; 4 Faculty of Medicine for Girls, Al-Azhar University, Cairo 11765, Egypt; dr.rehabomran@yahoo.com; 5 College of Applied Medical Sciences, Taibah University, Madinah 42353, Saudi Arabia; azza.abdallah@yahoo.com; 6 Faculty of Medicine, Menoufia University, Menoufia 32511, Egypt; 7 College of Medicine, Menia University, Menia 61519, Egypt; 8 Al Rayan College of Medicine, Medina 42541, Saudi Arabia; samy196327@gmail.com; 9 College of Medicine, Al Faisal University, Riyadh 11533, Saudi Arabia; yelmehallawy@alfaisal.edu; 10 Department of Medicine, King Salman Medical City, Medina 42316, Saudi Arabia; n.aldeeb@hotmail.com; 11 Department of Medicine, Bahçeşehir University, Istanbul 34353, Turkey; yaraisk98@gmail.com

**Keywords:** peak bone mineral density, nutrition, vitamin D, physical activity, metabolic equivalents, childbearing period

## Abstract

This is a comparative multicenter cross-sectional study that evaluated the potential determinants of Z-scores among premenopausal Saudi women before and after the age of peak bone density. The Study concluded that for better BMD among premenopausal women, attention should be paid to early physical activity and healthy nutrition, especially vitamin D, during the childbearing period. Objective: To explore the potential determinants of Z-scores among premenopausal Saudi females in different age groups before and after the expected age of peak bone density (PBD). Methods: This multicenter comparative cross-sectional study was conducted in Madinah and Jeddah, Saudi Arabia, between August 2021 and March 2022. We recruited 886 premenopausal females (605 (68.3%) below and 281 (31.7%) at or above the age of 30). The structured pre-coded Arabic questionnaire included sociodemographic data, a BMD questionnaire, menstrual history, an Arab Teen Lifestyle Study questionnaire, and food frequency data. Metabolic Equivalents (METs) were calculated from physical activity. Analysis of serum PTH, 25(OH) vitamin D (VD) was performed with chemiluminescent immunoassay. BMD was measured with a calcaneal qualitative ultrasound. Results: Most women had age-matched Z-scores, with very few (24 (2.7%)) being non-age-matched with no identified secondary causes. Significant Z-score determinants before PBD were BMI (OR: 0.167, *p* = 0.003) and total METs (OR: 0.160, *p* < 0.005). After the age of PBD, significant predictors were parity (OR: 0.340, *p* = 0.042), history of vitamin D deficiency (OR: 0.352, *p* = 0.048), and BMI (OR: 0.497, *p* = 0.019). Conclusions: Early determinants of Z-scores among premenopausal women were the nutritional status and physical activity. After the age of PBD, parity and vitamin D status offer additional determinants. For better BMD, attention should be paid to early physical activity and healthy nutrition, especially for vitamin D, with intensification of efforts during the childbearing period.

## 1. Introduction

In healthy adolescents, the maximum achievable bone mineral density (BMD), the peak bone mass (PBM), is usually attainable by the late teenage [1], with possible slight gains from ages 20 to 29 years [1]. Epidemiological research has shown that a 10% absolute rise in PBM can delay osteoporosis onset in later life by over a decade [2]. Conversely, a decrease of 6.4% in PBM is associated with a two-fold increase in fracture risk during adulthood [3].

Several factors influence PBM, including genetic, nutritional, and environmental factors. Early malnutrition, inadequate exercise, decreased dietary calcium intake, medications, smoking, and excess alcohol consumption may affect the achievability of the PBM [4,5]. Females can lose up to 10% of their bone mass during pregnancy and lactation, with a recovery period of 12 months [6]. Low BMD is associated with a lower incidence of fractures in premenopausal women than in older females due to a lower risk of falling, relative increase in muscle mass, and higher estrogen levels [7,8]. However, premenopausal women with a known secondary cause of osteoporosis have been found to have a high prevalence of bone fragility [5]. It is necessary to evaluate premenopausal women who have low BMD, even in the absence of fragility fractures, to prohibit the secondary causes of bone loss [7].

Although there is a similarity between T- and Z-scores in young individuals, the International Society for Clinical Densitometry (ISCD) recommends using Z-scores, which compare a young woman’s BMD to an age-, gender-, and ethnicity-matched population [8]. The ISCD considered a BMD Z-score of ≤2.0 in young women below the expected range for age [8]. Moreover, they recommended using two age periods: before and after the age of PBM during the interpretation of BMD readings among premenopausal females [8].

Published data on osteoporosis prevalence in Saudi Arabia among elderly men and postmenopausal women are increasingly alarming, ranging from 38.3–47.7% in the western region [9], 23.5% from the central region [10], and 37.4% from the eastern region [11]. In a hospital-based study, more than half of postmenopausal women were osteoporotic [12]. Therefore, the Saudi Arabia National Plan for Osteoporosis Prevention and Management in 2018 recommended conducting prospective multicenter studies to measure the incidence and risk factors of osteoporosis among Saudi women [13]. The preventive strategy should start with exploring the early determinants of PBM. Thus, the aim of this study was to investigate the clinical and biochemical factors that determine Z-scores among Saudi premenopausal women in two age groups: before and after the age of PBD.

## 2. Methods

### 2.1. Study Setting and Design

A comparative cross-sectional study was conducted in Madinah and Jeddah, Saudi Arabia, between August 2021 and March 2022.

### 2.2. Sample Size

The target population included healthy premenopausal adult Saudi females 20 to 44 years old. This age range was selected based on the expected age of natural menopause, around 50 years. The sample size is estimated using G*Power software (version 22), taking into consideration using an F-test, a linear multivariate regression analysis with a fixed model, a small effect size of 0.02, an alpha error probability of 0.05, and a power of 80%. A minimum required sample of at least 822 participants was estimated to achieve statistical power. The studied population was selected using a non-randomized consecutive convenient technique via campaigns in university campuses and large city malls.

### 2.3. Exclusion Criteria

Pregnant, lactating, or postmenopausal (defined as amenorrhea for 12 months) women were excluded. Exclusion criteria also included those with known secondary causes of osteoporosis (inflammatory diseases such as inflammatory bowel disease and rheumatoid arthritis), endocrine disorders such as Cushing’s syndrome, hyperparathyroidism, hyperthyroidism, type 1 diabetes, and hypogonadism), chronic obstructive pulmonary disease, diseases related to nutritional deficiencies such as celiac disease, cystic fibrosis, anorexia nervosa; and Subjects on medications suppressing ovulation or affecting bone remodeling (examples: corticosteroids, hormonal contraceptives, and antiepileptic) were excluded. Participants who gave incomplete or incoherent answers were excluded.

### 2.4. Data Collection

A pre-coded, structured, self-reported questionnaire was available as an electronic Google form to collect the participants’ data in the Arabic language. The questionnaire included sociodemographic data (age, education, occupation, and marital status). A modified version of the “Menstrual history questionnaire” was used for clinical data [14], in addition to a BMD questionnaire, which we adopted from Brockville General Hospital [15]. Lifestyle data collection was based on The Arab Teen Lifestyle Study (ATLS) questionnaire for physical activity [16], the “Compendium of Physical Activity for Youth” [17], and the “Short non-quantitative Food Frequency Questionnaire” [18].

### 2.5. Measurement of Body Mass Index

The body mass index (BMI) was calculated using the following equation: BMI = weight (kg)/height (m)^2^.

### 2.6. Bone Mineral Density Measurement

BMD measurements were performed on one heel for all women with the quantitative ultrasound (QUS) technique using Lunar Achilles Insight TM—GE Healthcare, which is a heel water-bath ultrasound system. The heel of the independent foot was placed between two ultrasonic transducers in a water bath at 37 °C. The ultrasound generates high-frequency sound waves to measure the heel BMD. T-scores and Z-scores were recorded using the manufacturer reference range after entering the subject’s age into the machine software, which would then match the T- and Z-score results according to age. Measurement results were displayed as a standard BMD fracture risk colored graph, and the stored and absolute values were printed. Daily quality *assurance* was carried out using the quality phantom. Due to the participants’ young age, we considered Z-score >−2 to be normal. We also used T-scores to assess bone mineral density, and we categorized T-scores into three categories: >−1, (−1–2.5), and <−2.5 [19].

### 2.7. Blood Sample Collection

Blood samples were collected under complete aseptic conditions. Samples were left in a plain tube for 30–60 min to allow spontaneous clotting at room temperature, then centrifuged at 3000× *g* for 10 min for serum separation. The obtained sera were frozen immediately at −70 °C for later analysis. Serum vitamin D was measured using a chemiluminescent microparticle immunoassay (ARCHITECT, Abbott, Chicago, IL, USA). Vitamin D deficiency is defined as a 25(OH)D below 20 ng/mL, insufficient at 21–29 ng/mL, and sufficient at 30–100 ng/mL [20]. Serum parathyroid hormone was measured using a chemiluminescent immunoassay technique (Beckman Coulter, Brea, CA, USA, ACCESS immunoassay systems).

### 2.8. Ethical Considerations

The study was performed in compliance with the Helsinki Declaration, and the study protocol was approved by the Ethics Committee of the Institutional Review Board (IRB) at Taibah University, College of Medicine, Ethical Committee No. TU-20-016. Written informed consent was obtained from all subjects and/or their legal guardian (s) before the study. The research objectives were written in the informed consent. The details of communication with the researchers were also mentioned in the informed consent form. All obtained data were kept confidential with the principal investigator.

### 2.9. Statistical Analysis

**Figure 1:**
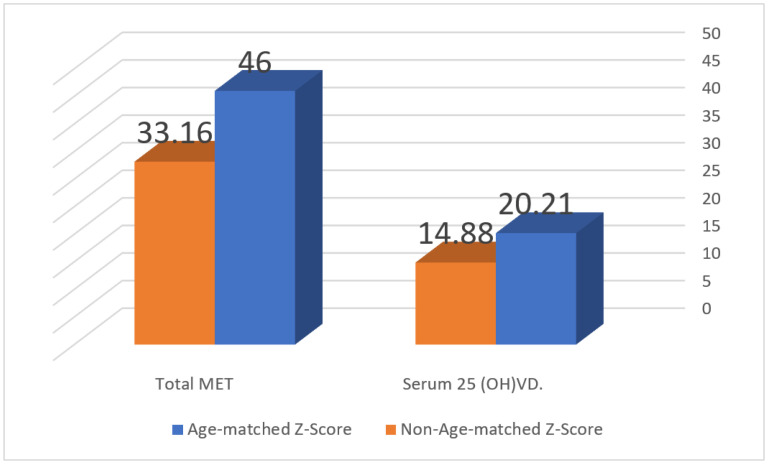
Significant differences between the age-matched Z score and non-age-matched group regarding total metabolic equivalents and serum 25(OH) VD.

The data were analyzed using the Statistical Package for the Social Sciences (IBM Corp. Released 2013, IBM SPSS Statistics for Windows, Version 22.0. Armonk, NY, USA). Figure 1 was constructed using a Microsoft Excel worksheet. Categorical variables were represented as percentages and frequencies, while numerical variables were summarized by calculating the median and interquartile range (IQR). The 886 females were divided into two groups: ≤30 and >30 years, based on the expected age of the PBM. Chi-square and non-parametric Man Whitney U tests were used for comparison between diverse groups. Multiple linear regression was used to determine the independent predictors of Z-scores before and after the age of PBM. All results considered the level of statistical significance at a *p* value of <0.05.

## 3. Results

One thousand Saudi females were screened, and 68 were excluded (23 had no data, 10 did not perform QUS measurements, 18 were pregnant or lactating, 11 were under corticosteroid therapy for various reasons, and 6 had collagen vascular diseases). Finally, the study included 886 premenopausal females (605 (68.3%) below and 281 (31.7%) above the age of 30).

The median age of the participants was 24 (IQR 12) years, ranging from 20 to 44 years. About 24.5% had irregular menstruation, 64.7% were single, and 31.2% had previous labor.

A positive family history of osteoporosis was elicited in 15.9%, while a history of a traumatic fracture was recorded at 11.7% with no reported cases of low trauma fragility fracture.

A previous diagnosis of vitamin D deficiency was present in 44.8%. The young age group showed significantly lower parity (*p* < 0.001), lower family history of osteoporosis (*p* < 0.05), and lower BMI (*p* < 0.001) as compared to the older age group. They also showed significantly more irregularities in their menstruation (*p* < 0.001) than the other group. The demographic characteristics and clinical features of both groups are summarized in Table 1.

The median serum 25(OH)D level was insufficient (22.8; IQR 14.03) in more than three-quarters of the participants (77.2%). A sufficient level was seen in only 142 females (22.8%), with no significant difference between both age groups (*p* = 0.656). However, parathyroid hormone (*p* < 0.01) levels were higher in females above the age of PBD (*p* < 0.01) (Table 1).

The median Z-score for all participants was 0.0 (IQR 1.5). The age-matched Z-score was reported in 97.3%, with no significant difference between the two age groups (*p* = 0.997). Only 24 (2.7%) females had non-age-matched Z-scores. The females with non-age-matched Z-scores were characterized by significantly lower levels of vitamin D (14.88 vs. 20.21 ng/mL), (*p* = 0.026), and total METs (33.16 vs. 46) (*p* = 0.019) compared to the age-matched females (Figure 1).

Females above the age of 30 showed significantly lower median Z-scores compared to the younger group (−0.2 vs. 0, *p* < 0.01, Table 1). The median T-score for osteoporosis in both groups was 0 (1.5). Most participants, 694 (78.3%), were at ≤1.0, 175 (19.8%) were between −0.1–2.4 at medium risk, and 17 (1.9%) were ≤2.5. Women above the age of 30 showed significantly lower median T-scores (−0.3 vs. 0, *p* < 0.001) compared to younger females (Table 1).

The lifestyle habits of participating females are summarized in Table 2. The median intake of dairy products for all participants was eight servings weekly. Only a few females (16.8%) received adequate dairy product servings, with non-significant variation between both age groups (*p* = 0.731). Females above 30 showed a significantly higher median weekly intake of fresh food (<0.01) and caffeinated drinks (*p* < 0.001) but a lower intake of salty food (*p* < 0.001). Smoking (shisha, cigarette, or combined) was reported by 31.2% of females; it was more prevalent among females above the age of 30 (64.1% vs. 14.1%, *p* < 0.001). The median daily screen time was 3 h; it was significantly longer in the younger age group (*p* < 0.001), but a sedentary life was significantly higher in older females (59.8% vs. 40.1%, *p* < 0.001).

Physical activity was reported among 70.2% of participants, with a total median METs of 45.2. The most physical activity practiced among females was walking (77.7%), followed by climbing upstairs (77.2%), household activities (57.9%), running (24.4%), and moderate-intensity sports (12.3%). Young females showed significantly higher METs than the older group for all physical activities except for household activities, which is significantly higher in the older group (*p* < 0.001) (Table 3).

Multiple linear regression analyses were performed to identify determinants of Z-scores in all participating women. The model was based on most clinical and laboratory variables, and all the assumptions were met. There was a normal distribution of error terms without autocorrelation between residuals (the Durbin-Watson statistic was 1.986) or collinearity. The Variance Inflation Factor (VIF) was <4. The overall model explains only a 3.4% variation of the Z-scores, but it is overall significant (F [18, 353] = 1.722, *p* = 0.034). BMI (OR: 0.179, *p* < 0.001) and total METs (OR: 0.149, *p* < 0.01) were significant predictors for all participants (Table 4).

Similarly, in the younger group, the overall model explains 3.6% of the Z-score, and it was significant (F [18, 317] = 1.687, *p* < 0.05). BMI (OR: 0.167, *p* = 0.003) and total METs (OR: 0.160, *p* < 0.005) were significant predictors of Z-scores (Table 4).

For older women, another multiple linear regression model was fitted based on the combination of independent variables, including the age of menarche, previous labor, menstrual irregularities, vitamin D deficiency, family history of bone fracture, BMI, natural vitamin D food sources, serum vitamin D, and parathyroid hormone. The overall model explains 30% of the Z-score (F [12, 24] = 2.294, *p* < 0.05). Parity (OR: 0.340, *p* = 0.042), vitamin D deficiency (OR: 0.352, *p* = 0.048), and BMI (OR: 0.497, *p* = 0.019) were significant predictors of the Z-scores (Table 4).

## 4. Discussion

In this study, most premenopausal females had age-matched Z-scores. Females with non-age matched scores showed significantly lower levels of METs and vitamin D. Early determinants of Z-scores were the nutritional status as reflected by BMI and the METs of physical activity. After the age of PBM, the drivers of Z-scores were BMI, parity, and vitamin D deficiency.

In our results, METs generated from different physical activities were independent significant predictors of Z-scores, especially in the young age group. Physical activity has been known for its significant impact on bone health if started early during growth and development [21]. Mechanical signals during physical activity trigger biochemical changes that augment bone turnover with more bone decomposition [21]. The beneficial effect of early physical activity might continue throughout life, as seen in different studies [22,23]. Thus, it is recommended that children and young adults should have daily high-intensity sports of at least 40 min with an element of overload, along with other activities such as running, jumping, or walking [24].

BMI has been found to have a favorable impact on bone mineral density in many previous studies, and it is accepted that increased body weight imposes mechanical loading, which contributes to increases in bone mass [25,26]. Madeira et al. [27] reported that a lean body mass rather than a fatty body mass was a predictor for BMD.

Years of menstruation have been shown to positively impact BMD, as long-lifetime exposure to endogenous estrogen supports bone formation and bone growth [28,29]. Pregnancy-associated long-term effects on BMD depend on several factors during and after delivery, such as breastfeeding and postpartum amenorrhea. Maternal bone loss of about 10% during pregnancy is found to be reversible within 6–12 months postpartum [30]. Increased parity has also been considered to negatively affect BMD [31,32]. Therefore, recent pregnancy and lactation must be taken into consideration during the interpretation of BMD measurement in premenopausal females.

Our results showed that both age groups had two different dietary patterns; younger females reported a significantly lower intake of legumes and fresh vegetables/fruit and a higher intake of salty food. This type of diet has unfavorable impacts on bone health and could, in part, explain our regression analysis results, which showed that the Z-scores of younger females were significantly related to their dietary patterns. Recent evidence suggests that the isoflavones content of legumes and soy intake may have a crucial role in preventing low BMD [33,34]. Therefore, the intake of a balanced diet of specific nutrients can be considered ideal for an effective preventive strategy for low BMD.

Our results showed that a history of previous vitamin D deficiency was an independent predictor of Z-scores of females aged >30, while a history of vitamin D consumption, calcium supplementation, and dairy food intake were not significant predictors in all our participants. Despite its proven effect in postmenopausal females, the benefits of vitamin D supplementation in premenopausal females are less clear [35]. One systematic review concluded that calcium from dairy products with or without vitamin D positively influences BMD in children as well as adults if their baseline calcium intake is low [36]. Vitamin D serum levels were insufficient in 77.2% of our participating women, with no significant difference between both groups. In a meta-analysis of randomized clinical trials, the association between vitamin D levels and BMD was unclear, with most of the results showing a positive correlation [37]. A position statement in 2019 highlighted a high prevalence of vitamin D deficiency in European and Middle Eastern countries, which may reach values up to 50% during the winter season [38].

Our results showed that females aged >30 had significantly lower Z-scores but higher parathyroid hormone levels compared to younger females. This may be due to previous exposure to vitamin D deficiency, which was more prevalent in older females. Secondary hyperparathyroidism takes years to improve serum calcium and phosphorous at the expense of bone health [39]. This can partly explain our finding of naturally occurring vitamin D food as the only food that remained in our significant predicting model in this older age group.

As recommended by ISCD [8], we relied on Z-score measurements to examine the BMD of healthy premenopausal Saudi females before and after achieving PBM. The majority (97.3%) showed age-matched Z-scores. Despite strict exclusion criteria of possible secondary causes, we reported 24 females (2.7%) with non-age-matched Z-scores equally distributed among both age groups, which represented a clinically meaningful low BMD. Relying on T-scores, we identified more cases (21.7%) with low scores (19.8% ≤ −1.0–−2.4 and 1.9% ≤ −2.5), with a higher prevalence among older females (25.8% vs. 16.8% for scores ≤ −1.0–2.4). This older age group showed significantly lower Z-scores and more risk factors, including a positive family history of osteoporosis, smoking, less physical activity, and higher parathyroid hormone levels when compared to the younger age group. Similar studies from Saudi Arabia reported higher rates of low BMD. A study from Madinah [40] reported a 3% rate of osteoporosis and 6% osteopenia, while other studies reported much higher rates (24% osteopenia and 11.9% osteoporosis [41], and 30.1% osteopenia and 6.5% osteoporosis [24,42]. Dual-energy X-ray absorptiometry (DEXA) is the preferred golden standard tool for the assessment of BMD. A recent Saudi study of females between the ages of 20 and 40 reported a low T-score in 19% of the participants using DEXA, the golden standard test [43]. Evaluation of low BMD in premenopausal women is challenging. About 0.5% of healthy young women have low T scores, reaching cut-off values of osteoporosis and 16% within the range of osteopenia diagnosis [44]. Therefore, for premenopausal females without a history of fragility fracture, the diagnosis of osteopenia or osteoporosis should not be solely based on BMD cut-off values. To diagnose primary osteoporosis in this age group, a non-age matched Z-score of ≤−2 should be associated with no identifiable secondary causes after extensive workup [45]. One study included women aged 20 to 44 years with established osteoporotic fractures and reported secondary causes in 90% of them [45].

## 5. Strengths and Limitations

The present study provides new insights regarding the determinants of decreased BMD before and after the age of PBM in premenopausal Saudi females. However, it does have some limitations. First, the cross-sectional design using the non-randomization technique limited the causal-effect relationship between the Z-score and its determinants, and the result cannot be generalized. Second, the use of QUS. However, many studies considered QUS as a reliable screening method for low BMD and recommended its use in primary care settings [46]. In this study, QUS was used in conjunction with the assessment of risk factors, which allowed for improved identification of females at risk. Third, the absence of a standardized Saudi database for QUS BMD limited a reliable interpretation of the individual BMD data. Fourth, the collected data on food frequency intake without estimating portion sizes or considering food fortification or supplementation may lead to measurement error. However, we attempted to improve the value of the data by considering bone-specific food items (caffeinated, natural sources, dairy products, salty food, and legume seeds).

## 6. Conclusions and Recommendations

Both physical activity and nutritional status represent the most significant predictors of the Z-score before the age of PBM. Later, parity and vitamin D deficiency, in addition to the nutritional status, appear to significantly affect the Z-scores among premenopausal females. Insufficient vitamin D is prevalent among premenopausal Saudi females of all age groups. It significantly and inversely affects Z-scores in females above the age of PBM. These findings necessitate introducing screening and prevention programs for vitamin D deficiency. This study highlights the need for public health awareness of factors helping in achieving good PBM and in maintaining bone density in later life, especially during the childbearing period. Public health strategies should include nutritional awareness, physical activity encouragement, and the avoidance of smoking and a sedentary lifestyle.

Very few premenopausal females had a clinically meaningful low Z-score with uncertain secondary causes. Diagnosis of primary osteoporosis in premenopausal females necessitates a thorough workup to ensure the absence of secondary causes and to provide evidence of excessive bone loss, disrupted bone microarchitecture, bone turnover, or demineralization. Therefore, further research should be directed to premenopausal women with altered bone metabolism with clinically meaningful low bone mass.

## Figures and Tables

**Table 1:** Demographic, clinical, laboratory, and Z-score characteristics of premenopausal females from two different age groups.

		Total N = 886	Females ≤ 30 Years N = 605 (68.3%)	Females > 30 Years N = 281 (31.7%)	*p* Value
Age: Median (IQR): Years		24 (12)	22 (4)	36 (8)	<0.001
Education	Below college education	335 (37.8%)	191 (32.3%)	144 (48.8%)	<0.001
	Undergraduate student	211 (23.8%)	197 (33.3%)	14 (4.7%)	
	College graduate	340 (38.4%)	203 (34.3%)	137 (46.4%)	
Occupation	Student	594 (67%)	580 (95.9%)	14 (5%)	<0.001
	Housewife	128 (14.4%)	3 (0.5%)	125 (44.5%)	
	Employee	164 (18.5%)	22 (3.6%)	142 (50.5%)	
Marital status	Single	533 (64.7%)	474 (88.3%)	59 (20.6%)	<0.001
	Married/previously married	291 (35.3%)	63 (11.7%)	228 (79.4%)	
Menarche: Median age (IQR)		13 (2)	13 (2)	13 (2)	0.231
Irregular menses		212 (24.5%)	167 (33.2%)	45 (17.8%)	<0.001
Parity	Nullipara	616 (71.1%)	541 (91.5%)	75 (25.4%)	<0.001
	Previous labor	270 (31.2%)	50 (8.5%)	220 (74.6%)	
Family history of bone fracture		101 (11.7%)	75 (14.7%)	26 (9.8%)	0.053
Family history osteoporosis		138 (15.9%)	80 (15.7%)	58 (21.8%)	<0.05
History of vitamin D deficiency		388 (44.8%)	253 (45%)	135 (50.6%)	0.882
History of vitamin D intake		326 (37.6%)	214 (41.6%)	112 (42.3%)	0.866
History of bone fracture		26 (11.5%)	15 (3.0%)	11 (4.3%)	0.334
BMI: Median (IQR)		23.6 (6.9)	22.9 (6.2)	26.35 (6.5)	<0.001
Parathyroid hormone: pg/mL		39.2 (32)	37.7 (28.9)	58.38 (53.69)	<0.01
Serum 25(OH) vitamin D: ng/mL		22.8 (14.03)	22.3 (16.2)	25.67 (27.54)	0.989
Insufficient vitamin D		482 (77.2%)	408 (77.6%)	74 (75.5%)	0.656
Sufficient vitamin D		142 (22.8%)	118 (22.4%)	24 (24%)	
Z-score: Median (IQR)		0 (1.5)	0 (1.5)	−0.2 (1.4)	0.01
Age-matched Z-Score		862 (97.3%)	575 (97.3%)	287 (97.3%)	0.997
Non-age-matched Z-Score		24 (2.7%)	16 (2.7%)	8 (2.7%)	
T score: Median (IQR)		0 (1.5)	0 (1.5)	−0.3 (1.4)	0.000
>−1		694 (78.3%)	481 (81.4%)	213 (72.2%)	0.006
−1–2.4		175 (19.8%)	99 (16.8%)	76 (25.8%)	
≥−2.5		17 (1.9%)	11 (1.9%)	6 (2%)	

Irregular menstrual periods (cycle-to-cycle variation of more than 20 days), Parity (a history of delivering at least one viable child as parity), BMI: Body Mass Index. IQR: Interquartile range.

**Table 2:** Dietary, smoking, and sedentary lifestyles among premenopausal Saudi females from two different age groups.

		Total N = 886	Females ≤ 30 N = 605	Females > 30 N = 281	*p* Value
Weekly food frequency: median (IQR)	Legume seeds	0 (1)	0 (0.25)	0.25 (1)	<0.001
	Dairy products	8 (9)	7 (10.5)	7 (8.3)	0.087
	Adequate daily dairy intake	149 (16.8%)	95 (16.1%)	54 (18.3%)	0.731
	Caffeinated drinks	8.5 (7.2)	8.25 (8.8)	9.0 (7)	<0.05
	Fresh vegetables/fruits	7 (6.8)	7 (6.8)	7.25 (9)	<0.01
	Salty food (pickles/junk)	2 (6)	2.1 (6)	2.0 (1.5)	<0.001
Smoking		276 (31.2%)	85 (14.4%)	191 (64.7%)	<0.001
TV/computer daily duration: median (IQR): HOURS		3.0 (3)	3.0 (2)	2.0 (3)	<0.001
Sedentary life (>2 h daily)		398 (44.9%)	301 (59.8%)	97 (40.1%)	<0.001

**Table 3:** Physical activities among the participating premenopausal Saudi females.

	Total N = 886		Females ≤ 30 Years N = 605		Females > 30 Years N = 281		*p* Value
Physical Activity	N (%)	MET: Median (IQR)	N (%)	MET: Median (IQR)	N (%)	MET: Median (IQR)	
Walking exercise	688 (77.7%)	1.75 (6.2)	467 (79%)	2.31 (7.42)	221 (74.9%)	1.65 (4)	<0.01
Ascending stairs	684 (77.2%)	32 (32)	476 (80.5%)	32.0 (48.0)	208 (70.5%)	16.0 (16)	<0.001
Running	216 (24.4%)	0 (2.6)	171 (28.9%)	0.00 (2.64)	45 (15.3%)	0.00 (0)	<0.001
Household activities	513 (57.9%)	1.16 (5.3)	333 (56.3%)	1.16 (3.5)	180 (61%)	2.31 (7)	<0.01
Bicycling	93 (10.5%)	0 (0)	76 (12.9%)	0.00 (0)	17 (5.8%)	0.00 (0)	<0.01
Swimming	73 (8.2%)	0 (0)	55 (9.3%)	0.00 (0)	18 (6.1%)	0.00 (0)	0.171
Moderate-intensity sport	109 (12.3%)	0 (0)	87 (14.7%)	0.00 (0)	22 (7.5%)	0.00 (0)	<0.01
Vigorous-intensity sport	81 (9.1%)	0 (0)	64 (12.8%)	0.00 (0)	17 (6.7%)	0.00 (0)	<0.05
Self-defense sport	28 (3.2%)	0 (0)	25 (5%)	0.00 (0)	3 (1.2%)	0.00 (0)	<0.05
Bodybuilding	112 (12.6%)	0 (0)	96 (19.1%)	0.00 (0)	16 (6.3%)	0.00 (0)	<0.001
Total	622 (70.2%)	45.2 (45.9)	416 (70.4%)	53.16 (48.3)	206 (69.8%)	35.38 (35.9)	<0.001

**Table 4:** Clinical, lifestyle, and laboratory predictors of Z-scores among healthy premenopausal females from different age groups.

	All Premenopausal Women N = 886			Premenopausal Women < 30 N = 605			Premenopausal Women > 30 N = 281		
Predictors	Odd Ratio	*p* Value	95% Confidence Interval	Odd Ratio	*p* Value	95% Confidence Interval	Odd Ratio	*p* Value	95% Confidence Interval
Age of menarche	0.044	0.400	−0.039–0.044	0.063	0.256	−0.032–0.119	−0.235	0.176	−0.296–0.057
Regularity of menstruation	0.060	0.252	−0.106–0.402	0.075	0.176	−0.082–0.447	−0.250	0.172	−1.601–0.302
Parity	0.034	0.523	−0.301–0.034	−0.004	0.937	−0.595–0.550	**0.340**	**<0.05**	**0.028–1.450**
History of vitamin D deficiency	0.043	0.438	−0.155–0.043	0.026	0.665	−0.214–0.334	**0.352**	**<0.05**	**0.007–1.437**
Family history of bone fracture	−0.014	0.799	−0.375–−0.014	−0.023	0.689	−0.427–0.282	0.207	0.193	−0.290–1.362
Family history osteoporosis	−0.035	0.525	−0.471–−0.035	−0.067	0.242	−0.636–0.161			
Weekly dairy products	−0.010	0.852	−0.023–−0.010	−0.007	0.909	−0.024–0.021			
Fresh food	0.070	0.186	−0.009–0.070	0.075	0.176	−0.009–0.049			
Salty food	−0.087	0.101	−0.052–−0.087	−0.087	0.122	−0.054–0.006			
Natural food sources of vitamin D	−0.023	0.683	−0.066–0.043	−0.063	0.283	−0.090–0.026	0.170	0.310	−0.064–0.192
Caffeinated drinks	0.053	0.329	−0.012–0.053	0.064	0.266	−0.011–0.040			
Serum Parathyroid hormone	−0.018	0.743	−0.006–−0.018	−0.029	0.611	−0.007–0.004	−0.116	0.497	−0.017–0.008
Serum vitamin D	0.004	0.944	−0.009–0.004	0.026	0.668	−0.008–0.013	−0.339	0.072	−0.050–0.002
**BMI**	**0.179**	**<0.01**	**0.016–0.179**	**0.167**	**0.003**	0.013–0.063	**0.497**	**0.019**	**0.018–0.186**
**Total METs**	**0.149**	**<0.01**	**0.001–0.149**	**0.160**	**0.005**	0.001–0.006			

**BMI:** Body Mass Index. **METs:** Metabolic Equivalents.

## Data Availability

The data used to support the findings of this study are available from the corresponding author upon request.

## Abbreviations

BMD	Bone Mineral Density
PBM	Peak Bone Mass
BMI	Body Mass Index
METs	Metabolic Equivalents
IQR	Interquartile Range
ISCD	International Society for Clinical Densitometry
QUS	Quantitative Ultrasound
